# Comprehensive Analysis of Bacterial Community Structure and Diversity in Sichuan Dark Tea (*Camellia sinensis*)

**DOI:** 10.3389/fmicb.2021.735618

**Published:** 2021-09-10

**Authors:** Kuan Yan, Linfeng Yan, Lina Meng, Hongbing Cai, Ailing Duan, Lian Wang, Quanzi Li, Ahmed H. El-Sappah, Xianming Zhao, Manzar Abbas

**Affiliations:** ^1^Faculty of Agriculture, Forestry and Food Engineering, Yibin University, Yibin, China; ^2^Key Laboratory of Sichuan Province for Refining Sichuan Tea, Yibin, China; ^3^Sichuan Province Tea Industry Group Co., Ltd., Yibin, China; ^4^State Key Laboratory of Tree Genetics and Breeding, Chinese Academy of Forestry, Beijing, China; ^5^Research Institute of Forestry, Chinese Academy of Forestry, Beijing, China; ^6^Genetics Department, Faculty of Agriculture, Zagazig University, Zagazig, Egypt

**Keywords:** bacterial diversity, high-throughput sequencing, Sichuan dark tea, pile-fermentation, qPCR

## Abstract

Bacteria and fungi present during pile-fermentation of Sichuan dark tea play a key role in the development of its aesthetic properties, such as color, taste, and fragrance. In our previous study, high-throughput sequencing of dark tea during fermentation revealed *Aspergillus* was abundant, but scarce knowledge is available about bacterial communities during pile-fermentation. In this study, we rigorously explored bacterial diversity in Sichuan dark tea at each specific stage of piling. Analysis of cluster data revealed 2,948 operational taxonomic units, which were divided into 42 phyla, 98 classes, 247 orders, 461 families, 1,052 genera, and 1,888 species. Certain members of the family *Enterobacteriaceae* were dominant at early stages of fermentation YC, W1, and W2; *Pseudomonas* at middle stage W3; and the highest bacterial diversity was observed at the final quality-determining stage W4. Noticeably, probiotics, such as *Bacillus*, *Lactobacillus*, *Bifidobacterium*, and *Saccharopolyspora* were also significantly higher at the quality-determining stage W4. Our findings might help in precise bacterial inoculation for probiotic food production by increasing the health benefits of Sichuan dark tea. This research also falls under the umbrella of the “Establish Good Health and Well-Being” Sustainable Development Goals of the United Nations Organization.

## Introduction

Tea can be considered a medicinal plant with a lot of health benefits, and it is consumed as a beverage across the world. China is the origin of the tea plant (*Camellia sinensis*) and has great importance in Chinese culture. In China, plentiful tea products are found, relying upon growth areas, processing techniques, and polyphenol oxidase contents (Jie et al., 2006; Lee et al., 2015). Dark tea is one of the six major tea types found in China, and it is cultivated in Sichuan, Yunnan, Guangxi, Hunan, and Hubei (Peng et al., 2014; Liu, 2016). Dried green tea leaves are the raw material that is processed to make dark tea *via* microbial fermentation and solid-state fermentation (SSF). Growth of microbes, such as bacteria and fungi, is a key step in the biosynthesis of dark tea during the wet piling technique, which determines the aesthetic value of dark tea, such as stale or mellow taste and reddish brown wine (Li et al., 2017).

Sichuan province is a prominent dark tea production area due to its unique and favorable climatic conditions for the growth of raw material and specialty of processing technology in pile-fermentation. It is hypothesized that the potential microbial resources of the Sichuan region are entirely different as compared with other dark tea production areas. Traditional bacterial culture techniques are employed for undermining structural variations among microbial communities in dark tea, in which only a few bacterial strains can be isolated because selective bacterial strains can grow on available mediums (Ge et al., 2019). The probability of fake results of microbial diversity in the fermentation of dark tea is significantly high by culturing techniques.

Contrastingly, culture-independent methods have the advantage over culture-dependent methods because of its potential for microbial community structure detection in any kind of medium even with low abundance. Recently, culture-independent methods are being widely used in the food industry to undermine the composition of microbes more accurately and comprehensively (Li et al., 2017). The principal of culture-independent methods is based on direct diversity analysis *via* sequencing of ribosomal RNA (rRNA) genes without any involvement of a culture-medium step; that is why it is robust, fast, and cost-effective and provides high resolution of microbial communities. However, culture-independent methods are scarcely applied to investigate the microbial community structure in dark tea (Chen et al., 2013; Fu and Liu, 2015; Zhang et al., 2016; Ge et al., 2019).

In this study, high-throughput sequencing was employed to undermine the entire diverse bacterial community structure and to specifically reveal dominant bacterial taxa at each stage of pile-fermentation present in Sichuan dark tea. Our study provides a preliminary exploration of the dynamic characteristics of the bacterial community during the piling process and reveals the actual modulator behind the development of the unique aesthetic values of Sichuan dark tea. A better understanding of the microbial community structure and their abundance at each aesthetic value-determinant stage paves the way to regulate their concentration and develop new techniques to precisely develop unique tastes and probiotic foods for human beings.

## Materials and Methods

### Experimental Materials

In order to conduct this research, samples of Sichuan dark tea were provided by the Sichuan Tea Industry Group Co., Ltd., and these were prepared from fresh leaves of tea plants collected in summer and autumn. During the fermentation process, the leaves of tea plants were mixed thoroughly to ensure homogeneity, and tap water was sprinkled in an adequate quantity to maintain 65–75% (w/v) solid contents and the temperature at 45–71°C. Samples were collected from tea piles at 15-day intervals and subjected to sensory evaluation as described by GB/T 23776-2009 (Gong et al., 2009). The fermentation process is over as the tea mass becomes reddish-brown and free of its stringent taste. Samples were collected during the fermentation process in triplicate and at the following time intervals; days 0 (YC), 8 (W1), 16 (W2), 24 (W3), and 32 (W4). The temperature of each fermentation tea pile was recorded from the center at the depth of 40 cm almost every day.

### DNA Extraction

For DNA extraction, 5 g of each sample was suspended in 50 mL sterile Tween-NaCl buffer [0.9% NaCl (w/v), 0.05% Tween 20 (v/v) and 2% polyvinylpolypyrrolidone (w/v)] and homogenized by mixing thoroughly for 30 min *via* sonication at 4°C. The solution was further passed through a sterile gauze and subsequently centrifuged at 2,000 rpm for 2 min at 4°C to remove course material. To collect the microbial communities, the supernatant was shifted into a new tube and centrifuged at 10,000 rpm for 10 min at 4°C. Microbial genomic DNA was extracted from the precipitate using the E.Z.N.A.^TM^ HP Plant DNA Kit (Omega Bio-Tek Inc., GA, United States) according to the manufacturer’s protocol. Finally, DNA was purified using the E.Z.N.A.^TM^ Soil DNA Kit to remove impurities, such as polyphenol, that may have a negative effect on the PCR reaction (Yan et al., 2021).

### PCR Amplification and Sequencing Analysis

The primer pair 338F: 5′-ACTCCTACGGGAGGCAGCAG-3′ and 806R: 5′-GGACTACHVGGGTWTCTAAT-3′ was employed to amplify the V3+V4 region of the bacterial 16S rRNA gene (Zeng et al., 2011; Apprill et al., 2015). The PCR reaction conditions were as follows: initial denaturation at 95°C for 3 min, denaturation at 95°C for 30 s, annealing at 55°C for 30 s, extension at 72°C for 45 s, 30 cycles in total; final extension was at 72°C for 10 min and held at 10°C for 10 min. The total PCR reaction system was 20 μL with the following ingredients; 4 μL of 5 × PCR buffer (with Mg^2+^), 2 μL of 2.5 mmol/L dNTP, 0.8 μL of 5 μmol/L P1 (338F), 0.8 μL of 5 μmol/L P2 (806R), 0.4 μL of 5 U/μL Taq Enzyme, 2 μL of DNA template, and 10 μL of ddH_2_O. PCR products of all the same samples were mixed together and electrophoresed by running on a 2% agarose gel. Gel cutting was performed with a sterile sharp edge blade, and subsequently, the AxyPrepDNA Gel Recovery Kit (AXYGEN^®^) was used for gel elution to recover the correct DNA band. Furthermore, PCR products with good quality were subjected to library construction and sequenced by using the robust Illumina MiSeq^TM^ PE300. Pair reads were merged into a single sequence by following the overlap relationship between PE reads and filtered for quality control to a avoid splicing effect. Finally, reads obtained by sequencing were checked for their quality, and sequence direction was corrected (Mukherjee et al., 2014; Yan et al., 2021). According to primer use, proper barcoding/labeling was performed on each step to avoid any error.

### Taxonomic Assessment and Data Analysis

The USEARCH manual version 7.1^1^ was deployed to extract non-repetitive sequences from optimized sequences, which is very useful to remove redundant calculations during data analysis^2^, and to remove non-repetitive single sequences^3^. Similarly, UPARSE software (see text footnote 1) was deployed for clustering analysis at a 97% similarity index to get representative sequences of operational taxonomic units (OTUs) (Ye et al., 2017). The RDP classifier Bayesian algorithm was deployed to perform taxonomic analysis of OTUs at the 97% similarity index. Bacterial community composition present in every dark tea sample was classified and counted at each level of phylogeny, such as kingdom, phylum, class, order, family, genus, and species (Chen et al., 2016).

To construct a rarefaction curve, a specific number of individuals from each sample were randomly selected to evaluate their OTUs, and a number of individuals with their relevant OTUs were used to construct the curve. The rarefaction curve is used to compare the abundance of each specie in given samples with variable sequencing reads and to validate the amount of sequencing data. Rarefaction analysis of OTUs at the 97% similarity index obtained from 16S rRNA gene sequence reads *via* illumina MiSeq platform was performed at the mothur website^4^, and the curve was made using the R tool (Amato et al., 2013). To evaluate the sequencing depth index, α-diversity, such as Chao 1, ACE, Shannon, and Simpson of each sample, were individually calculated, and the abundance as well as the diversity of microbial communities in dark tea were analyzed at different fermentation stages (Rogers et al., 2016). A Venn diagram was constructed to count the number of common and unique OTUs in all samples (Fouts et al., 2012). According to the taxonomic analysis results, single or multiple samples were compared at each classification level, and the R tool was employed to construct community structure component diagrams, histogram combined analysis diagrams, and RDA principal coordinate analysis diagram (Lu et al., 2016; Zhou et al., 2017).

### Statistical Analysis

All data is explained in mean values of standard deviation (SD) and analyzed by one-way analysis of variance (ANOVA). A Duncan multiple-comparison test was applied to detect variations among means of all samples at a *p*-value < 0.05 level of significance. All correlation and path coefficient analyses were performed with SPSS Statistics 20.0 (SPSS Inc., Chicago, IL, United States) and Excel 2019.

## Results

### Sequencing Data Analysis

We analyzed the diverse bacterial communities found in Sichuan dark tea at five different stages during the fermentation process by large-scale sequencing-based analysis of 16S rRNA gene sequences. The sequencing data of the bacterial colonies found in all samples collected at five different stages of fermentation are given in Table 1. The total number of sequencing reads of sample YC are 62,456, the total number of nitrogenous bases are 26,118,006 bp, and the average sequence length is 418.59 bp. Similarly, the total number of effective sequencing reads of sample W1 are 46,909, the total numbers of nitrogenous bases are 19,638,346 bp, and the average sequence length is 420.24 bp. Sequencing analysis revealed 40,399 full-length sequence reads in sample W2, the total number of nitrogenous bases is 17,285,142 bp, and the average read length is 427.75 bp. In sample W3; 43,362 full length sequencing reads were obtained with a total number of 18,361,869 bp nitrogenous bases, and the average read length is 423.51 bp. Finally, after elimination of raw and incomplete reads, 60,400 total reads were obtained in sample W4, containing 25,535,185 bp total number of nitrogenous bases, and the average length of sequence reads was 422.57 bp (Table 1).

**TABLE 1:** Sequencing analysis of 16S rRNA gene of bacterial communities in all five samples.

Sample	Reads	Total bases	Average length
YC	62,456	26,118,006	418.59
W1	46,909	19,638,346	420.24
W2	40,399	17,285,142	427.75
W3	43,362	18,361,869	423.51
W4	60,400	25,535,185	422.57

*Column 1 represents sample name, column 2 represents reads obtained after elimination of raw and incomplete reads, column 3 is comprised of total number of nitrogenous bases, and column 4 represents average read length in base pair.*

**FIGURE 1:**
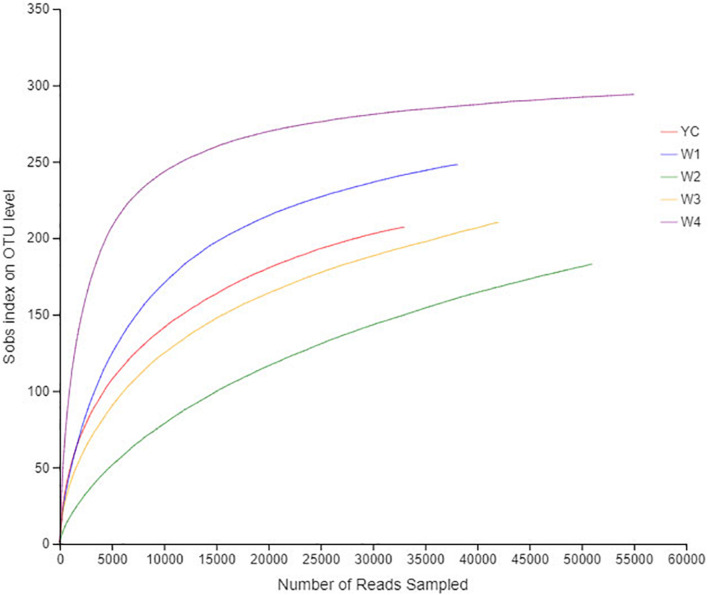
Rarefaction curves of all samples. The abscissa represents the amount of randomly selected sequencing data; the ordinate represents the number of species observed. The flat curve shows that the amount of sequencing data for this study is sufficient.

The rarefaction curve was drawn to show the sampling depth of each sample, which can be used to evaluate either sequencing date and is sufficient to represent all bacterial strains present in each sample. The flatness of each rarefaction curve representing all five samples proved that the sampling process had adequately collected the experimental material (Figure 1). The bacterial diversity in the amount of each randomly collected sample was enough to construct an individual DNA library for each sample. The confidence level about the amount of each bacterial community structure in Sichuan dark tea samples was very high to accurately reflect the bacterial community.

### OTUs Cluster Analysis

**FIGURE 2:**
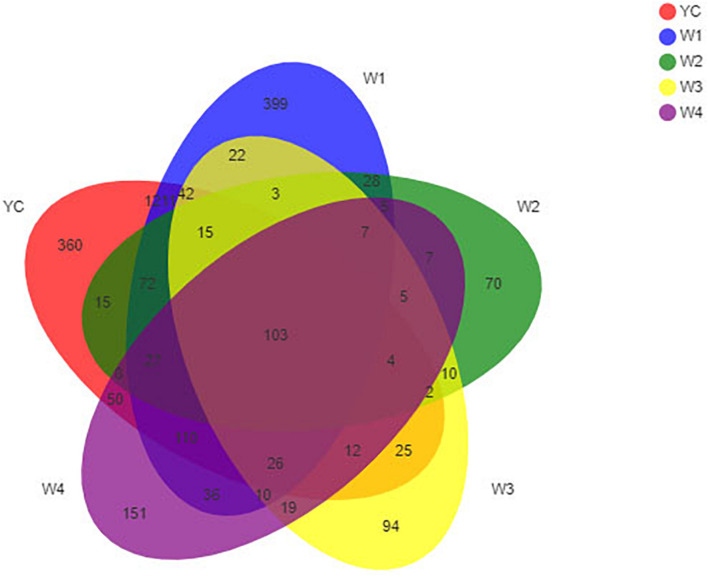
OTU Venn analysis. Different groups are represented by different colors, and numbers in overlapping portions represent the number of species common among all groups.

The bioinformatics and statistical analyses were performed at the OTU level with a 97% similarity index. Furthermore, a Venn diagram was constructed to display compositional similarity among the different bacterial communities present in all samples, and the overlapping of different samples was intuitively displayed at different classification levels. A total number of OTUs obtained by clustering the valid data were 2,948, and these were further divided into 42 phyla, 98 classes, 247 orders, 461 families, 1,052 genera, and 1,888 species (Supplementary Table 1). Similarly, OTUs in each stage were 2,082, 2,116, 381, 399, and 580, respectively. Among all samples, YC and W1 samples shared the highest number of 1,211 OTUs, YC and W2 samples shared 15 OTUs, YC and W3 samples shared 25 OTUs, and YC and W4 samples shared 50 OTUs. The unique OTUs that were not common or overlapping in each sample were 360 in YC, 399 in W1, 70 in W2, 94 in W3, and 151 in W4, which represented 17.3, 18.9, 18.4, 23.6, and 26.0% of total OTUs, respectively (Figure 2). The total number of common OTUs among all five samples collected at five different stages of pile-fermentation were 103, which were 3.6% of the total number of OTUs in all samples. These results indicate that the bacterial community structure of Sichuan dark tea has undergone significant changes during pile-fermentation.

### Microbial Abundance and Diversity Analysis

Bacterial community richness and diversity was explored in Sichuan dark tea samples collected at different stages of pile-fermentation. Noticeably, the sequencing coverage of all samples was higher than 0.9984, which represents the actual conditions of bacterial communities present in Sichuan dark tea samples collected at different stages of fermentation (Table 2). In general, the higher Shannon and lower Simpson indices represents the higher bacterial community diversity index in a sample (Huang et al., 2018). We observed the highest bacterial community diversity in Sichuan dark tea samples collected at YC and W4 stages, followed by the W1 period, and the lowest bacterial community diversity was observed in samples collected at the W2 stage.

**TABLE 2:** Bacterial community richness and diversity indices of Sichuan dark tea.

Sample	Shannon	Simpson	ACE	Chao	Coverage
W1	1.65	0.3649	287	284	0.9984
W2	0.78	0.6875	242	214	0.9988
W3	1.67	0.4025	260	267	0.9987
W4	2.47	0.1857	289	293	0.9995
YC	2.21	0.1854	329	315	0.9988

*First column consists upon sample name, columns 2–4 represent Shannon, Simpson, ACE and Chao 1 values of diversity index in each sample. Last column contains average of values of diversity index.*

To investigate the abundance of the bacterial community structure in Sichuan dark tea collected at different stages of fermentation, the Chao and ACE indices were measured. We observed the highest bacterial community abundance in dark tea samples collected at the YC and W4 stages, followed by samples collected at the W1 stage, and the lowest bacterial community abundance was observed in samples collected at the W2 and W3 stages. Our results show that the abundance and diversity of the bacterial community present in Sichuan dark tea samples was quite different at different stages of fermentation.

### Bacterial Community Structure Analysis

**FIGURE 3:**
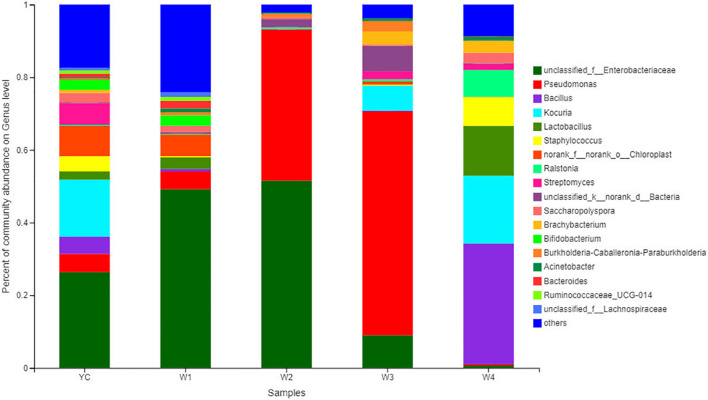
Bacterial community structure bar plot analysis at the genus level. The ordinate is the sample name, and the abscissa is the proportion of different taxa at the genus level in a sample. Different taxa are represented by columns with different colors, sizes, and proportion of species.

All Sichuan dark tea samples collected at different stages of pile-fermentation were analyzed to disclose the structure of the bacterial communities. Bacterial communities with higher abundance were annotated and clearly classified into 18 genera, and bacterial communities with relatively low abundance were merged together into a single category expressed by others (Figure 3). For example, bacterial communities with relatively high abundance at the genus level were *Pseudomonas*, unclassified_f_Enterobacteriaceae, *Bacillus*, *Kocuria*, *Ralstonia*, *Lactobacillus*, *Staphylococcus*, etc. (Figure 3). At the YC stage of fermentation, the significantly higher bacterial communities were unclassified_Enterobacteriaceae (26.38%), and the relatively high abundant bacterial communities at the genus level were *Kocuria* (15.72%), *Streptomyces* (5.92%), *Pseudomonas* (5.02%), *Bacillus* (4.73%), and *Staphylococcus* (4.19%).

**FIGURE 4:**
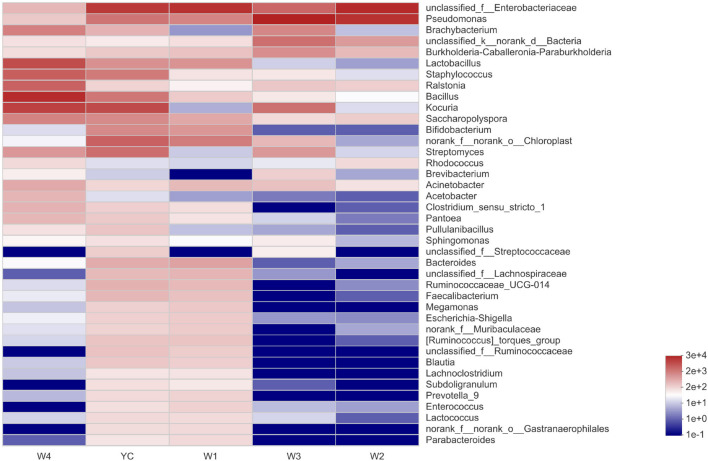
Community heat map analysis at the genus level. The abscissa represents the group name, and the ordinate represents the taxa name. Abundance changes of different taxa in each sample are displayed by color gradient of the color block. The bar on the right side of the figure represents the abundance value by color gradient.

At stage W1, the dominantly higher bacterial communities were unclassified_Enterobacteriaceae (26.38%), and the relatively high abundant bacterial communities at the genus level were *Pseudomonas* (4.91%), *Lactobacillus* (3.18%), *Bifidobacterium* (2.90%), and *Bacteroides* (2.10%). Similarly, the highest abundance of bacterial community at stage W2 was unclassified_ Enterobacteriaceae (51.55%), followed by *Pseudomonas* (41.50%), unclassified_Bacteria (2.15%), and the relative abundance of other bacterial genera were all less than 1%. *Pseudomonas* (61.61%) was an absolutely dominant bacterial strain at the W3 stage, and with relatively high abundance were unclassified_ Enterobacteriaceae (8.98%), *Kocuria* (6.86%), and unclassified_Bacteria (6.97%). At stage W4, the highest relative abundance was of *Bacillus* (33.20%), followed by *Kocuria* (18.63%), *Lactobacillus* (13.74%), *Staphylococcus* (7.92%), and *Ralstonia* (7.39%). We also observed that *Pseudomonas* and unclassified_Enterobacteriaceae displayed higher abundance at the YC, W1, W2, and W3 stages, and *Bacillus*, *Kocuria*, and *Lactobacillus* have higher abundance in the W4 period (Figure 4). Our results show that the bacterial diversity has obvious differences with the different piling-fermentation stages of Sichuan dark tea.

### Effect of Bacterial Community Composition on Active Ingredients

**FIGURE 5:**
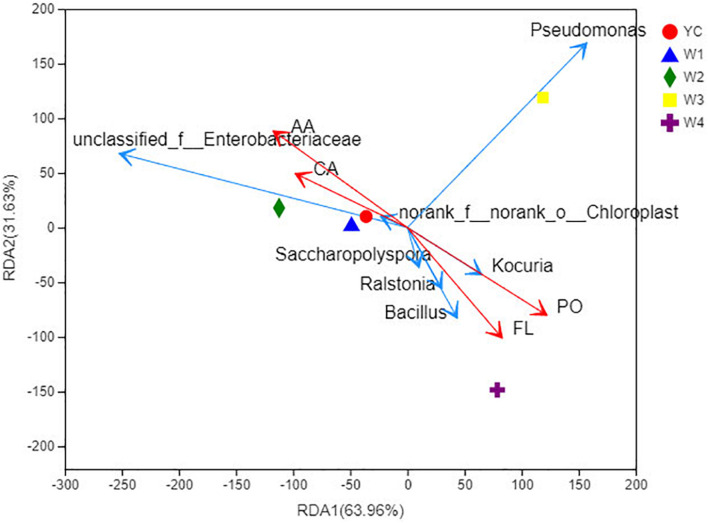
RDA at the genus level. Points of different colors or shapes represent sample groups under different environments or conditions in the figure; taxa are represented by green arrows in the RDA diagram; quantitative environmental factors are represented by red arrows. The length of the environmental factor arrow can represent the degree of influence of the environmental factor on the taxa data; the angle between the environmental factor arrow represents the positive and negative correlation (acute angle: positive correlation; obtuse angle: negative correlation; right angle: no correlation). Projection from the sample point to the arrow of the quantitative environmental factor, the distance between the projection point and the origin, represents the relative influence of the environmental factor on the distribution of the sample community. PO, polysaccharides; FL, flavonoids; CA, caffeine; AA, amino acids.

Redundancy analysis (RDA) is a type of PCA analysis that is constrained by environmental factors. For the pictorial representation of the relationship between bacterial flora at the genus level and environmental factors, samples and environmental factors were drawn on the same 2-D sequence diagram (Figure 5; Huhe et al., 2017). Among the four active compounds of Sichuan dark tea, polysaccharides have a positive correlation with total flavonoids, and caffeine has a positive correlation with amino acids. Furthermore, correlation was analyzed among the active compounds of dark tea, bacterial taxa, and different stages of pile-fermentation. We observed that effective formation of caffeine and amino acids predominantly exists at the following stages of fermentation: YC, W1, and W2.

Noticeably, the polysaccharides and total flavonoid contents were increased significantly during pile-fermentation stage W4. Similarly, the following bacterial genus *Bacillus*, *Ralstonia*, and *Saccharopolyspora* were significantly correlated with total flavonoid contents, and *Kocuria* was closely related with the formation of polysaccharides. The dominant bacterial genus at stage W3 was *Pseudomonas*, which displayed no relationship with any of the abovementioned four active ingredients of dark tea. These results prove that the biosynthesis of active ingredients of Sichuan dark tea were significantly affected by the composition of the bacterial community found in pile-fermentation.

## Discussion

The time required to accomplish the pile-fermentation cycle of dark tea and development of flavor, color, fragrance, and nutritional value are predominantly determined by the composition and fermentation stage-specific variations in the microbial community present in the fermentation reaction (Chai et al., 2015). Better understanding of the microbial community in the piling process is a prerequisite for precise manipulation of microbial flora to improve the aesthetic value of Sichuan dark tea. On the other hand, traditional culturing methods to explore microbial flora in dark tea samples is time-consuming, labor-intensive, and unsustainable because all bacterial species cannot be cultivated. We employed robust high-throughput Illumina Miseq^TM^ sequencing technology, which revealed 253,526 high-quality bacterial sequence reads with an average length of 422.53 bp. Furthermore, the clustering of valid sequencing data revealed 2,948 OTUs at a 97% identity threshold of 16S rRNA, which were classified as 42 phyla, 98 classes, 247 orders, 461 families, 1,052 genera, and 1,888 species. These findings lead us to conclude that bacterial flora present in the fermentation process are a result of inoculation from tea leaves (epiphytic and endophytic microbes) and the surrounding environment.

In recent years, only fungi have been the focus of researchers exploring the composition of the microbial community in dark tea, such as *Eurotium cristatum* (Yan et al., 2021), but knowledge about the bacterial community is scarce (Zeng et al., 2020). Bacterial flora displayed a lagging growth phase at the early flowering stage of *Camellia sinensis* due to the exponential growth of *E. cristatum*, which transformed into bacterial exponential growth at the late flowering stage to establish a symbiotic relationship with *E. cristatum* (Liu et al., 2014). Noticeably, the bacterial flora present in the dark tea of different regions is unique (Zhao et al., 2017). In Sichuan dark tea, two relatively abundant bacterial genera were unclassified_f_Enterobacteriaceae and the *Pseudomonas* sequence analyzed during the YC to W3 stages of fermentation. Certain members of the *Enterobacteriaceae* family retain disease-causing potential, encompassing beneficial commensal microbiota opportunistic pathogens that can inflict considerable morbidity and mortality on compromised hosts, and a few are harmless and are widely distributed in the environment, including soil, water, plants, insects, and animals (Ito et al., 2019); that is why members of this genus were abundantly found (51.55%) during the early stages of pile-fermentation (W2). *Pseudomonas*, *Vibrio*, and *Staphylococcus* are generally considered to be conditional pathogens in dark tea (Williams et al., 2010). Comparatively, *Pseudomonas* was higher, 61.61%, in the dark tea samples collected at the W3 stage, and further studies are invited to explore its specific role.

The highest diversity in the bacterial community was observed at the quality-determining W4 stage of Sichuan dark tea. For example, *Bacillus* was highest, 33.20%, at the W4 stage, probably due to the decrease in temperature, which shows that the composition of the bacterial community during pile-fermentation is temperature-dependent (Endres et al., 2011). We can control the distribution of the bacterial community by regulating the temperature during dark tea pile-fermentation and, ultimately, the quality of dark tea. For example, the quick drying of dark tea is performed at 50°C during the final stage, W4, and at which abundance of *Bacillus* is also highest. The genus *Bacillus* are probiotics that have beneficial health effects on the intestines of animals and humans, play a role in the frequency and characteristics of feces, and skin properties (Endres et al., 2011). For example, *B. coagulans* is a health beneficial food ingredient, included in the qualified safety presumption (QPS) list by the European Food Safety Agency (2008), and approved by the United States Food and Drug Administration (Pozen et al., 2011) recognized safety standards (GRAS) (Zhao et al., 2013). Because the probiotic bacterial strain *B. coagulans* is dominant during the pile-fermentation process of dark tea, it can also be employed to improve the flavor and shelf life of different foods.

Other probiotic genera, *Lactobacillus* and *Bifidobacterium*, were also abundantly found at the final stage, W4, and these are also found in the human intestine and are widely used in the production of various types of fermented foods (Handjiev and Kuzeva, 2018). For example, the relative abundance of *Lactobacillus* was higher (13.74%) at the final stage, W4, and it helps in digestion, is beneficial for human and animal intestines, and is being widely used in the biosynthesis of yogurt and sausage (Cavalheiro et al., 2019). *Lactobacillus* present in black tea is also beneficial for intestinal flora and the health of mice (Arellano et al., 2020; Gao et al., 2020). The share of *Saccharopolyspora* was 2.85% at the W4 stage, which is an antitumor agent (Liu et al., 2005); therefore, it can be used in food processing to increase the health benefits of dark tea. Additionally, Butenyl-spinosyn is produced by *Saccharopolyspora*, which is a strong insecticidal agent with a broad pesticidal spectrum (Rang et al., 2021).

Biological active compounds present in Sichuan dark tea, such as polysaccharides, flavonoids, caffeine, and amino acids, are highly correlated with the abundance of bacterial genus during pile-fermentation. During the process of pile-fermentation, the concentration of these biologically active components declined due to microbial metabolic activities, and our findings are in accordance with Pu’er tea (Li et al., 2018). Similarly, cellulase, hemicellulase, and protease enzymes secreted by microbes during metabolic activities also have a catalytic effect on the main metabolites (Abe et al., 2008). Further studies are invited to unravel the relationship between biologically active ingredients of dark tea and the microbial diversity during the pile-fermentation process of Sichuan dark tea so that the quality of dark tea can be improved.

## Conclusion

Sichuan dark tea is economically important due to its unique aroma, and it has been prepared *via* pile-fermentation for decades. Microbes play a crucial role in the development of its unique aroma, nutritional value, and physical characteristics. In-depth knowledge about piling stage-specific bacterial composition will provide benchmarks for precise inoculation of probiotic bacteria for biofortification. High-throughput sequencing of the bacterial 16S rRNA gene revealed that certain members of the family *Enterobacteriaceae* were dominantly present in early piling stages YC, W1 and W2; *Pseudomonas* was dominant at stage W3; and the highest bacterial diversity was at stage W4. We observed that probiotic bacterial genera, such as *Bacillus*, *Lactobacillus*, *Bifidobacterium*, and *Saccharopolyspora* were in abundance at the final piling stage W4. In conclusion, precise inoculation of members of these bacterial genera in dark tea might improve its nutritional value and health benefits.

## Data Availability Statement

The datasets presented in this study can be found in online repositories. The names of the repository/repositories and accession number(s) can be found below: European Nucleotide Archive, accession no: PRJEB46897.

## Author Contributions

KY, MA, AE-S, and XZ: conceptualization. KY and MA: writing—original draft. KY, LY, LM, AD, LW, AE-S, and HC: drawing figures. XZ, QL, AE-S and MA: editing and proofreading. KY and XZ: supervision. KY: project administration. All authors contributed to the article and approved the submitted version.

## Conflict of Interest

LY and HC were employed by company Sichuan Province Tea Industry Group Co., Ltd. The remaining authors declare that the research was conducted in the absence of any commercial or financial relationships that could be construed as a potential conflict of interest.

## Publisher’s Note

All claims expressed in this article are solely those of the authors and do not necessarily represent those of their affiliated organizations, or those of the publisher, the editors and the reviewers. Any product that may be evaluated in this article, or claim that may be made by its manufacturer, is not guaranteed or endorsed by the publisher.

## Footnotes

1
http://drive5.com/uparse/

2
http://drive5.com/usearch/manual/dereplication.html

3
http://drive5.com/usearch/manual/singletons.html

4
https://mothur.org/wiki/miseq_sop/
